# Risk factors for lymph node metastasis and surgical scope in patients with cN0 non-small cell lung cancer: a single-center study in China

**DOI:** 10.1186/s13019-021-01695-5

**Published:** 2021-10-18

**Authors:** Bu Jianlong, Zhang Pinyi, Wu Xiaohong, Zhao Su, Pang Sainan, Ning Jinfeng, Xu Shidong

**Affiliations:** 1 Department of Thoracic Surgery, Harbin Medical University Cancer Hospital, Nangang District, Harbin, 150081 China; 2 Department of Anesthesiology, Harbin Medical University Cancer Hospital, Harbin, China

**Keywords:** Non-small cell lung cancer, NSCLC, Lymph node metastasis, Ground-glass opacity, GGO

## Abstract

**Background:**

It is difficult to determine the lymph node metastasis of patients with clinically negative lymph nodes (cN0) non-small cell lung cancer (NSCLC) before surgery. The purpose of this study is to investigate risk factors of lymph node metastasis in cN0 NSCLC, thereby to identify the surgical indications for lymph node dissection in cN0 NSCLC.

**Methods:**

We conducted a retrospective study of patients with tumor size ≤ 30 mm who underwent radical resection of NSCLC. Binary logistic regression analysis was applied to predict risk factors for lymph node metastasis, and subject operating characteristics (ROC) curve was used to evaluate the independent risk factors.

**Results:**

Overall, 44 patients (6.8%) with cN0 NSCLC had lymph node metastasis. Factors of tumor consolidation diameter (p < 0.001) and preoperative serum carcinoembryonic antigen (CEA) level (p = 0.017) are independent risk factors lymph node metastasis in cN0 NSCLC. The ROC curve showed that the cut-off value of consolidation diameter was 16.5 mm, and the area under the curve (AUC) was 0.825 (p < 0.001, 95% CI 0.780–0.870); the cut-off value of serum CEA level was 1.765 μg/L, and the AUC was 0.661 (p < 0.001, 95% CI: 0.568–0.754). Moreover, 8 of 461 patients with tumor parenchyma ≤ 16.5 mm had lymph node metastasis, and 36 of 189 patients with tumor parenchyma > 16.5 mm had lymph node metastasis.

**Conclusion:**

Tumor consolidation diameter and preoperative serum CEA are independent factors to predict cN0 NSCLC with tumor size ≤ 30 mm. For patients with tumor parenchyma > 16.5 mm, the probability of lymph node metastasis is higher and lymph node dissection is recommended. For patients with tumor parenchyma ≤ 16.5 mm, the probability of lymph node metastasis is lower and lymph node sampling is feasible.

## Introduction

Lung cancer has become the most common malignant tumor in China with the highest morbidity and mortality, of which non-small cell lung cancer (NSCLC) accounts for 80% [1, 2]. The overall 5-year survival rate of lung cancer is 16%, and the 5-year survival rate of early-stage lung cancer patients after timely treatment can be increased to 50% [3]. Recent developments in imaging modalities and the widespread application of low-dose helical computed tomography (CT) for lung cancer screening have led to an increase in the detection rate of early-stage lung cancer. Ground-glass opacity (GGO) is a slightly increased density on high-resolution computed tomography (HRCT) lung window, in which the bronchial and vascular textures are still visible [4]. Some patients with early-stage lung cancer appear as GGO on CT, and patients with GGO-based early-stage lung adenocarcinoma have been reported to have a favorable prognosis after surgery [5]. The current standard method for early NSCLC surgery is lobectomy combined with complete lymph node dissection or sampling [6]. However, researches show that there are fewer cases of lymph node metastasis in GGO-based early-stage NSCLC [7, 8]. Most early-stage NSCLC has no suspicious lymph node metastases and is identified as clinically negative lymph nodes (cN0) NSCLC. Therefore, whether patients with cN0 NSCLC need lymph node dissection or sampling has become a focus of attention.

In the past few decades, various biomarkeres have been explored for the prognosis of early stage NSCLC [9, 10]. The predictive role of systemic inflammatory laboratory parameters, such as neutrophil to lymphocyte ratio (NLR), platelet to lymphocyte ratio (PLR), and lymphocyte to monocyte ratio (LMR), have also been confirmed in patients with NSCLC [11, 12]. However, whether these inflammatory laboratory parameters have a predictive effect on the metastasis of NSCLC lymph nodes has been rarely reported.

This study aims to investigate the factors that can predict cN0 NSCLC lymph node metastasis, including basic characteristics of patients, imaging characteristics and preoperative examination of some hematological indicators. We selected cN0 NSCLC patients with tumor diameter ≤ 30 mm and without imaging metastatic lymph nodes before surgery to study the incidence and risk factors for lymph node metastasis, thereby to provide the surgical indications for lymph node dissection in cN0 NSCLC.

## Methods

### Patients

A retrospective analysis was performed by using data collected from the clinical database of the department of thoracic surgery in Harbin Medical University Cancer Hospital. The study cohort was composed of consecutive patients scheduled to undergo initial surgery for NSCLC from January 2018 to December 2019. The inclusion criterias in this study were: (1) histopathologically confirmed primary NSCLC; (2) chest high-resolution CT (HRCT) was performed before operation; (3) the maximum diameter of tumor ≤ 30 mm; (4) lobectomy and systematic lymph node dissection had been performed; (5) blood cell analysis and serum carcinoembryonic antigen (CEA) test were performed before operation. Patients were excluded with the following conditions: (1) histopathologically confirmed carcinoma in situ; (2) history of neoadjuvant chemotherapy for NSCLC before operation; (3) history of other malignant tumors; (4) preoperative chest CT showed mediastinal lymphadenopathy or hilar lymphadenopathy; (5) distant metastasis cannot be excluded. Ultimately, 650 patients were enrolled into the present study. This study was approved by the Institutional Ethics Review Board of Harbin Medical University Cancer Hospital. Informed written consent was obtained from all the patients before the study.

### Preoperative examinations

All the patients received pretreatment assessments including detailed clinical history, physical examination, a series of biochemical blood tests, and imaging examinations including magnetic resonance imaging (MRI) or CT of head, chest HRCT, upper abdominal ultrasound or CT, emission computed tomography (ECT), supraclavicular lymph node ultrasound and cardiopulmonary function test. The NSCLC staging was based on the 8th edition of TNM classification.

### Operations

All the patients in this study underwent single-port video-assisted thoracic surgery, and had no history of lung surgery or second surgery. If intraoperative frozen pathological results showed that the lung tumor was malignant (non-small cell lung cancer), whether it was a peripheral nodule or a deep lesion, anatomical lobectomy and systematic lymph node dissection were performed. In our center, the surgical scope of systemic lymph node dissection included stations 2–4 and stations 7–14 lymph nodes for right lung tumors, and stations 4–14 for left lung tumors. N2 was defined as stations 2–9 lymph nodes and N1 was defined as stations 10–14 lymph nodes. The status of lymph node involvement was defined as pN0 (pathologically confirmed no lymph node metastasis), pN1 (pathologically confirmed any metastatic lymph node in stations 10–14) and pN2 (pathologically confirmed any metastatic lymph node in stations 2–9).

### Pathology

Tumor histological types were recorded by two experienced pathologists. Histopathological analyses were performed according to WHO criteria (5th edition) [13]. Intraoperative pathological types were divided into adenocarcinoma, squamous cell carcinoma and other types of tumors (including large cell carcinoma, carcinoid, atypical carcinoid, poorly differentiated carcinoma and adenosquamous carcinoma, etc.). All resected lymph nodes, including mediastinal lymph nodes, hilar lymph nodes and intrapulmonary lymph nodes, needed to be pathologically examinated after surgical dissection.

### Data collection

The information we collected mainly included general information of patients, preoperative imaging examinations, laboratory examinations and histopathological results. The general information of patients included age, gender and smoking history; preoperative imaging examinations included maximum tumor diameter, maximum tumor consolidation diameter, tumor location and consolidation/tumor (C/T) ratio shown by chest HRCT; preoperative laboratory examinations included NLR, PLR, LMR and serum CEA levels. Histopathological results included intraoperative histopathological types, postoperative histopathological types and lymph node pathological results.

Patients were divided into two groups according to the age. One group was younger than 60 years old (age ≤ 60) and the other group was older than 60 years old (age > 60). The tumor size was defined as the maximum dimension of the tumor on lung window. The consolidation size was defined as the maximum dimension of the solid component on lung window excluding GGO. The C/T ratio was defined as the maximum dimension of consolidation on lung window setting divided by the maximum dimension of the tumor on lung window setting. Pulmonary nodules were described as pure GGO (C/T ratio = 0), mixed GGO (0 < C/T ratio < 1) and solid nodules (C/T ratio = 1).

### Statistics

All data analysis applications are SPSS 18 software (SPSS, Inc., Chicago, IL, USA). Risk factors for lymph node metastasis were analyzed by Student’s t-tests, Kolmogorov–Smirnov tests(K-S tests), χ^2^ tests, Fisher’s exact test, and logistic regression. The cut-off values of serum CEA level and consolidation size were determined by the receiver operating characteristic (ROC) curve. The statistical significance was considered when p < 0.05.

## Results

### Patients general information

A total of 650 patients who had initial surgery for NSCLC were included in this study. There were 398 men and 252 women, with a mean age of 58.3 years (range, 30–82 years) at the first diagnosis. 186 (186/650 = 28.6%) patients had a history of smoking. The general characteristics of the patients are listed in Table 1.

**Table 1:** General characteristics and their relationship with lymph node metastasis (χ2-test and Fisher’s exact test)

Variables	Total	pN0	pN1 + N2	χ^2^	p
All patients	650	606 (93.2%)	44 (6.8%)		
Gender				1.595	0.207
Female	398	375 (94.2%)	23 (5.8%)		
Male	252	231 (91.7%)	21 (8.3%)		
Age, years				0.794	0.435
< 60	342	316 (92.4%)	26 (7.6%)		
≥ 60	308	290 (94.2%)	18 (5.8%)		
Smoking history				8.439	0.004
None	464	441 (95.0%)	23 (5.0%)		
Yes	186	165 (88.7%)	21 (11.3%)		
Pathological type				0.951	0.633^※^
Adenocarcinoma	601	561 (93.3%)	40 (6.7%)		
Squamous cell carcinoma	36	33 (91.7%)	3 (8.3%)		
Other types of malignant tumors*	13	12 (92.3%)	1 (7.7%)		
Tumor locations				6.247	0.178^※^
Right upper lobe	243	232 (95.5%)	11 (4.5%)		
Right middle lobe	53	48 (90.6%)	5 (9.4%)		
Right lower lobe	99	92 (92.9%)	7 (7.1%)		
Left upper lobe	183	169 (92.3%)	14 (7.7%)		
Left lower lobe	72	65 (90.3%)	7 (9.7%)		

pN0: Pathological diagnosis without lymph node metastasis. pN1 + N2: Pathological diagnosis with N1 and/or N2 metastasis

*The “other types of malignant tumors” patients included 2 patients with undifferentiated carcinoma, 5 patient with carcinoid, 1 patient with large cell carcinoma and 5 patient with adenosscale squamous cell carcinoma. ^※^Fisher's exact test is applied to the data

### Frequency and distribution of lymph node metastasis

A total of 11,455 lymph nodes were removed from 650 patients, with an average of 17.6 lymph nodes removed per patient. There were 606 cases (606/650 = 93.2%) without lymph node metastasis, 44 cases (44/650 = 6.8%) with lymph node metastasis, 19 cases (19/650 = 2.9%) with only N1 metastasis, 12 cases (12/650 = 1.8%) with only N2 metastasis, and 13 cases (13/650 = 2.0%) with both N1 and N2 metastasis. In the 12 patients with single N2 metastasis, there were 3 patients who had the smallest consolidation size of 10 mm, and the tumors were all located in the upper lobe of the left lung (Patient One had one lymph node metastasis in station 6, Patient Two had one lymph node metastasis in station 6 and one lymph node in station 9, and Patient Three had one lymph node metastasis in station 5 and one lymph node metastasis in station 6). In the 44 cases with lymph node metastasis, there were 17 cases (17/650 = 2.62%) with the lymph node metastasis in the lung lobe (including station 12, 13, 14). There were 74 cases (74/650 = 11.4%) of pure GGO patients, 239 cases (239/650 = 36.8%) of solid nodules, and 337 cases (337/650 = 51.8%) of mixed GGO patients. There was no lymph node metastasis in patients with pure GGO, 9 cases (9/337 = 3.8%) of mixed GGO had lymph node metastasis, and 35 cases (35/239 = 14.6%) of solid nodule had lymph node metastasis.

### Risk factors of lymph node metastasis

The chi-square test and Fisher’s exact test were applied to analyze the relationship between patient’s age, gender, smoking history, lesion location and intraopeative pathological type and lymph node metastasis. These results show that smoking history (χ2 = 8.439, p = 0.004) is related to lymph node metastasis (Table 1). T test was applied to analyze the relationship between the tumor size (t = − 6.24, p < 0.001) and consolidation size (t = − 10.80, p < 0.001) and lymph node metastasis, suggesting that the two factors are both related to lymph node metastasis (Table 2). K-S test was used to analyze the relationship between C/T ratio, serum CEA level, NLR, PLR and LMR and lymph node metastasis. These results suggest that C/T ratio (z = 3.082, p < 0.001) and serum CEA level (z = 1.851, p = 0.002) are related to lymph node metastasis (Table 3).

**Table 2:** General characteristics and their relationship with lymph node metastasis (Student’s t-test)

Variables	pN0	pN1 + N2	t	p
Tumor size (mm)	17.13 ± 6.41	22.41 ± 5.34	− 6.24	< 0.001
Consolidation size (mm)	10.94 ± 8.33	20.82 ± 5.64	− 10.80	< 0.001

pN0: Pathological diagnosis without lymph node metastasis. pN1 + N2: Pathological diagnosis with N1 and/or N2 metastasis

**Table 3:** General characteristics and their relationship with lymph node metastasis (K-S test)

Variables	pN0	pN1 + N2	Z	p
C/T ratio	0.65 (0.28, 1.00)	1.00 (1.00, 1.00)	3.082	< 0.001
Serum CEA level (μg/L)	1.90 (1.19, 3.02)	2.83 (1.67, 6.19)	1.851	0.002
NLR	1.63 (1.27, 2.19)	1.79 (1.20, 2.29)	3.082	0.748
PLR	116.14 (92.57, 150.60)	114.45 (82.96, 170.80)	0.677	0.133
LMR	5.11 (3.86, 7.90)	5.03 (3.43, 6.21)	0.984	0.287

pN0: Pathological diagnosis without lymph node metastasis. pN1 + N2: Pathological diagnosis with N1 and/or N2 metastasis. C/T ratio: consolidation size/tumor size ratio. CEA: carcinoembryonic antigen. PLR: platelet-to-lymphocyte ratio. NLR: neutrophil-to-lymphocyte ratio. LMR: lymphocyte-to-monocyte ratio

**Fig. 1:**
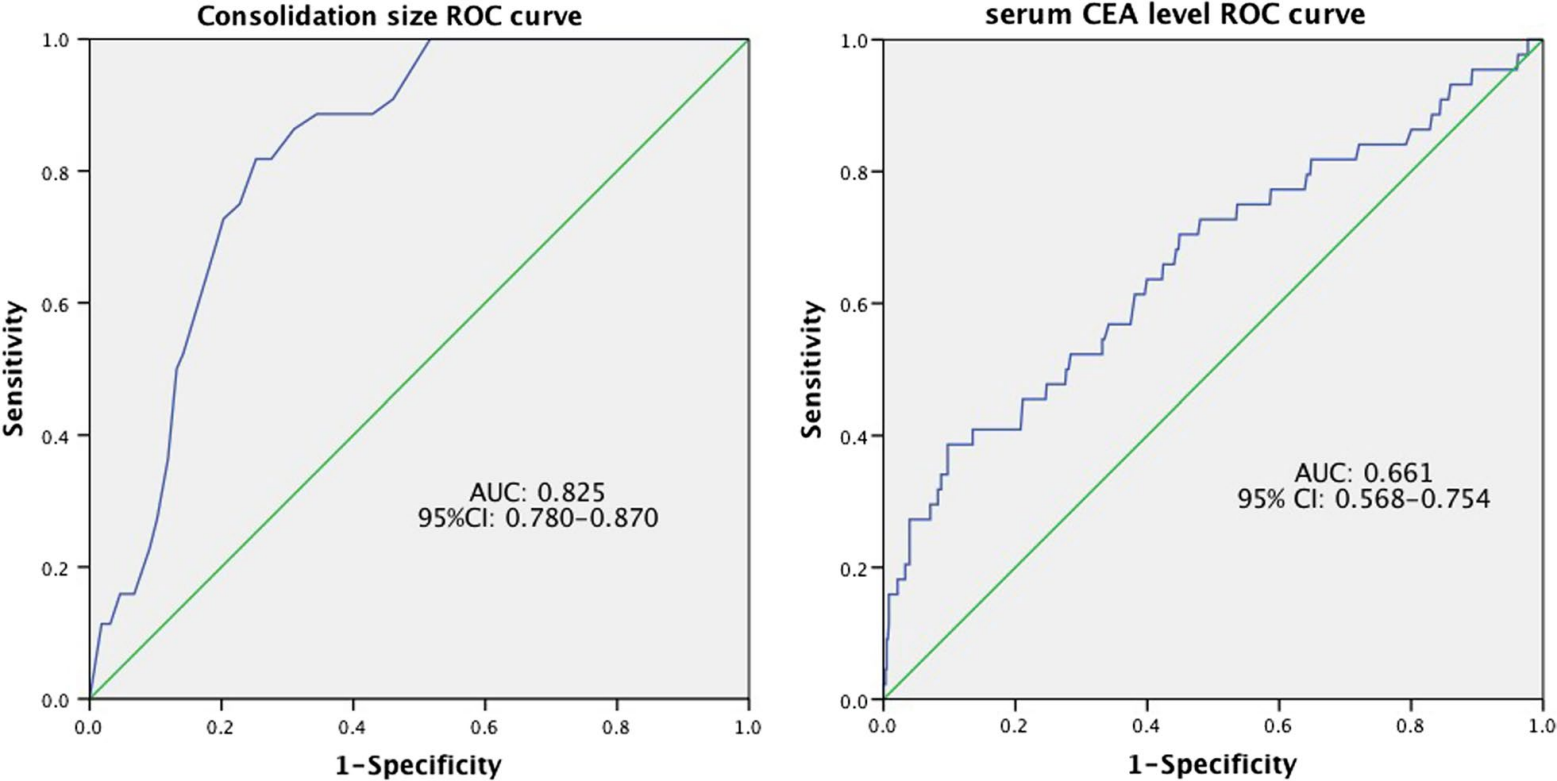
Receiver operating characteristic (ROC) curve of consolidation size and serum CEA level values in predict lymph node metastasis. The AUC for consolidation size was 0.825 (p < 0.001, 95% CI 0.780–0.870) with a sensitivity of 81.8% and specificity of 74.8%. The AUC for scrum CEA level was 0.661 (p < 0.001, 95% CI 0.568–0.754) with a sensitivity of 75% and specificity of 46.4%

The relationships between smoking history, tumor size, consolidation size, C/T ratio, serum CEA level and lymph node metastasis were analyzed by binary logistic regression. The results show that consolidation size [odds ratio (OR) = 1.142, p < 0.001] and serum CEA level (OR = 1.091, p = 0.017) are independent risk factors of lymph node metastasis. Moreover, the possibility of lymph node metastasis increases with the consolidation size and the CEA value (Table 4). The ROC curve was used to analyze the consolidation size and serum CEA level (Fig. 1). The results show that the consolidation size of 16.5 mm is the best cut-off point, the area under the curve (AUC) was 0.825 (p < 0.001, 95% CI 0.780–0.870), with a sensitivity of 81.8% and a specificity of 74.8%; serum CEA level of 1.765 μg/L is the best cut-off point, the AUC was 0.661 (p < 0.001, 95% CI 0.568–0.754), with a sensitivity of 75% and a specificity of 46.4%.

**Table 4:** Independent risk factors of lymph node involvement by multivariate analysis

Variables	Odds ratio	95% CI	p
Consolidation size	1.142	1.094–1.193	< 0.001
Serum CEA level	1.091	1.015–1.172	0.017

*CI* confidence interval

There were 189 patients with tumor consolidation size > 16.5 mm including 36 patients (36/189 = 19.0%) having lymph node metastasis. 461 patients with tumor consolidation size ≤ 16.5 mm, of which 8 cases had lymph node metastasis (8/461 = 1.7%).

## Discussion

Although lobectomy plus lymph node dissection or sampling is still the standard surgical method for NSCLC [6], studies have shown that stage IA NSCLC and GGO-based lung adenocarcinoma have no diffenrences in survival time between the lobectomy group and the sublobectomy group [8, 14]. These findings indicate that the tumor of patients with cN0 NSCLC can be completely removed by sublobectomy, which may achive a similar prognosis to the procedure of lobectomy as well. The study by Suzuki K et al. showed that for peripheral pulmonary nodules that were less than 2 cm and dominated by ground glass, with sufficient margins (the study definition was at least 5 mm), sublobectomy has close to 100% of 5-year recurrence-free survival and the surgical method should be recommended [15]. In fact, sublobectomy inevitably leads to some of the lymph nodes in the lung lobes that cannot be removed. To some extent, it can be concluded that the probability of N1 metastasis is extremly low in cN0 NSCLC [16]. According to the JCOG 0802 study published at the 101st AATS annual meeting, the peripheral suspicious NSCLC nodules (maximum tumor diameter ≤ 2 cm, solid component/tumor [CTR] > 0.5) had a higher local recurrence rate after segmentectomy than lobe resection. Eguchi et al. showed that spread through air spaces (STAS) positive T1N0M0 lung adenocarcinoma sublobectomy had a higher risk of locoregional recurrences and subsequent lung cancer-specific deaths than lobectomy [17]. For the early stage NSCLC, whether to perform sublobectomy still needs more comprehensive data support.

Study by Suzuki K et al. indicated that patients with cN0 NSCLC who showed negative mediastinal lymph node enlargement (metastasis) in preoperative imaging examination, still appeared to have mediastinal lymph node metastasis in postoperative pathology reports [18]. Similarly, our study found that 25 patients (25/650 = 3.8%) who did not prompt mediastinal lymph node metastasis before surgery had N2 metastasis. In consistent with the study by Wang et al. [19] reporting 1.5% of single N2 metastasis, our fingdings suggest that N2 metastasis may appear in patients with cN0 NSCLC, and the absence of N1 metastasis cannot completely exclude N2 metastasis. Alternatively, it can be reckoned that patients with cN0 NSCLC may have N2 metastasis without N1 metastasis.

Mediastinal lymph node dissection can remove mediastinal lymph nodes and clarify the stage of lung cancer, providing a basis for the formulation of postoperative treatment plans for patients. However, the mediastinal lymph node dissection would not only prolong the operation time, but also bring additional surgical risks [20]. Studies have confirmed that there are fewer cases of lymph node metastasis in pure GGO or GGO-based lung cancer [8]. Thus, if the method of screening patients with potential lymph node metastasis without increasing trauma can be applied before surgery, the surgical trauma to patients without lymph node metastasis could be reduced, and it may be a guide for clinicians to select surgical methods.

In this study, we found that the consolidation size and preoperative serum CEA level were independent risk factors of lymph node metastasis. Our results suggest that the frequency of lymph node metastasis increases with the consolidation size of tumor, which is consistent with the results by Murakawas et al. [21]. Thus, the factor of consolidation size might be more accurate to predict lymph node metastasis. To some extent, it also confirms that the T-stage measurement of consolidation size in the 8th edition of TNM staging may be more accurate for tumor staging. Using ROC curve analysis for serum CEA level, the AUC was 0.661 but the accuracy rate was not very ideal. In the ROC curve, we found that when the consolidation size was 9.5 mm, it was the maximum predictive value with 100% sensitivity, and the specificity at this point was 48.3%. The patients included in this study were not found lymph node metastasis when the consolidation size was less than 9.5 mm, while just one study found N1 metastasis in 5 mm solid nodule [22]. Thus, it is difficult to find an absolute boundary of consolidation size to predict lymph node metastasis in NSCLC. In this research, no lymph node metastasis was found in those with pure GGO, which is consistent with the results of study by Zha et al. [23]. Until now, systematic lymph node dissection emphasizes the dissection of mediastinal lymph nodes (N2) and hilar lymph nodes. Currently, the clinical postoperative pathological results may not show all the metastastic lymph nodes in lung lobes. Thus, we did not discuss the relevant factors of intrapulmonary lymphatic metastasis. However, a complete N1 (especially the lymph nodes in the lung lobe) dissection would play a critical role in sublobectomy for cN0 lung cancer.

Immune cells, such as neutrophils and lymphocytes, with tumor cells and stromal cells constitute the microenvironment of tumor, and play an important role in tumor development and progression [24, 25]. In addition, an experimental study showed that all tumor associated immune cells (except MUM1 + cells) in stage III tumor specimens were significantly higher than those in stage I specimens [26]. We tried to detect whether NLR, PLR, LMR and other indicators could predict lymph node metastasis. Although the statistical results are not significantly correlated, it cannot be ruled out that other immune cell or blood test indicators could predict lung cancer lymph node metastasis.

Despite of the present findings, this study still possesses some limitations. Firstly, our clinical database of cN0 NSCLC, as a single-center administrative database, cannot capture every subtle factor, some of which may be critical for clinicians. Thus, further multi-center randomized trials are needed to verify these results. Secondly, we had little information on disease-free survival because of the lack of recurrence records based on long-term follow-up, which failed to evaluate the prognostic significance of lymph node dissection. Thirdly, further investigation for us will also involve the pathways and mechanisms of NSCLC lymph node metastasis before strict indications for prophylactic lymph node dissection for cN0 NSCLC are formally defined.

## Conclusion

In conclusion, the present study demonstrates that consolidation size and serum CEA level are independent predictors of lymph node metastasis of cN0 non-small cell lung cancer with tumor size ≤ 30 mm. While the tumor consolidation diameter has a high accuracy in predicting lymph node metastasis, and the preoperative serum CEA level has a low accuracy in predicting lymph node metastasis. Patients with consolidation size > 16.5 mm are recommended to receive lymph node dissection due to higher probability of lymph node metastasis. For patients with NSCLC with consolidation size ≤ 16.5 mm, lymph node sampling should be performed due to lower probability of lymph node metastasis.

## Data Availability

Not applicable.

## Abbreviations

AUC: Area under the curve
CEA: Carcinoembryonic antigen
ECT: Emission computed tomography
GGO: Ground-glass opacity
HRCT: High-resolution computed tomography
LMR: Lymphocyte to monocyte ratio
MRI: Magnetic resonance imaging
NLR: Neutrophil to lymphocyte ratio
NSCLC: Non-small cell lung cancer
PLR: Platelet to lymphocyte ratio
ROC: Subject operating characteristics
